# Hiding in Fresh Fruits and Vegetables: Opportunistic Pathogens May Cross Geographical Barriers

**DOI:** 10.1155/2016/4292417

**Published:** 2016-02-16

**Authors:** Zahra S. Al-Kharousi, Nejib Guizani, Abdullah M. Al-Sadi, Ismail M. Al-Bulushi, Baby Shaharoona

**Affiliations:** ^1^Department of Food Science & Nutrition, College of Agricultural and Marine Sciences, Sultan Qaboos University, P.O. Box 34, Al-Khod, 123 Muscat, Oman; ^2^Department of Crop Sciences, College of Agricultural and Marine Sciences, Sultan Qaboos University, P.O. Box 34, Al-Khod, 123 Muscat, Oman; ^3^Department of Soils, Water and Agricultural Engineering, College of Agricultural and Marine Sciences, Sultan Qaboos University, P.O. Box 34, Al-Khod, 123 Muscat, Oman

## Abstract

Different microbial groups of the microbiome of fresh produce can have diverse effects on human health. This study was aimed at identifying some microbial communities of fresh produce by analyzing 105 samples of imported fresh fruits and vegetables originated from different countries in the world including local samples (Oman) for aerobic plate count and the counts of Enterobacteriaceae,* Enterococcus*, and* Staphylococcus aureus*. The isolated bacteria were identified by molecular (PCR) and biochemical methods (VITEK 2). Enterobacteriaceae occurred in 60% of fruits and 91% of vegetables.* Enterococcus* was isolated from 20% of fruits and 42% of vegetables.* E. coli* and* S. aureus* were isolated from 22% and 7% of vegetables, respectively. Ninety-seven bacteria comprising 21 species were similarly identified by VITEK 2 and PCR to species level.* E. coli*,* Klebsiella pneumoniae*,* Enterococcus casseliflavus*, and* Enterobacter cloacae* were the most abundant species; many are known as opportunistic pathogens which may raise concern to improve the microbial quality of fresh produce. Phylogenetic trees showed no relationship between clustering of the isolates based on the 16S rRNA gene and the original countries of fresh produce. Intercountry passage of opportunistic pathogens in fresh produce cannot be ruled out, which requires better management.

## 1. Introduction

Being sources of high energy and rich in minerals, vitamins, fibers, and phenolics, fruits and vegetables constitute an important food group that has been linked to maintenance of well-being of individuals [1] and to reduced incidence of some chronic diseases [2]. In addition to the nutritional value of fresh produce, their diverse microbiomes can pass through stomach to the gut where they establish specific associations with the host resulting in various effects on human health [3]. Recently, interesting relationships have been found between gut microbiota and obesity, malnutrition, cancer, personal motivation, and decision-making in which microbial balance is critical for maintaining the healthy state [3, 4].

On the other hand, the increased consumption of fruits and vegetables in recent years has been found to be accompanied by an increase in the number of human infections and outbreaks [5] as these can serve as reservoirs for pathogens or opportunistic pathogens [3]. Fruits and vegetables can be contaminated with spoilage or pathogenic bacteria at any stage from production to consumption [6, 7]. Although their microflora is dominated by spoilage bacteria, yeasts, and molds, fruits and vegetables can harbor pathogenic bacteria such as* Salmonella*,* Escherichia coli*,* Bacillus cereus*,* Campylobacter* spp.,* Yersinia enterocolitica*,* Listeria monocytogenes*, and* Clostridium botulinum*, as well as some viruses and parasites [6]. In Royal Hospital, Oman, May 2008,* B. cereus* caused a nosocomial outbreak with gastroenteritis and affected 58 individuals.* B. cereus* and its toxin were found in different foods including vegetables [8]. A more serious outbreak, May–July 2011, was caused by Shiga toxin producing* E. coli* O104:H4 in Germany where 2987 cases of gastroenteritis, 855 cases of hemolytic-uremic syndrome, and 53 deaths were reported. Fenugreek sprouts were found to be contaminated with the causative agent [9]. Bean sprouts were linked to two outbreaks in USA, in 2014; one was caused by* L. monocytogenes* in August while* Salmonella* Enteritidis caused the other one just a month later.* L. monocytogenes* was also involved in another outbreak that was linked to caramel apples in October 2014 in USA [10]. Opportunistic pathogens can cause life-threatening infections mainly in immunocompromised people but they may have positive effects on the health of immunocompetent individuals by stimulating immune functions and priming the immune system continuously [3]. Nonpathogenic microbes associated with fruits and vegetables may have various consequences on the quality of the produce by affecting the rate of the food spoilage. Fruits and vegetables seem also to be the sources that disseminate many microbes to food preparation areas [11].

In Oman, large quantities of fruits and vegetables are imported nearly from all around the world to provide a year-round supply [12] to this country which is located at the cross-roads of the exchange of cultivated plants [13]. This study aims at assessing the microbial load of some fresh fruits and vegetables imported into or grown in Oman and to identify the isolated bacteria by biochemical and molecular methods with emphasis on emergent opportunistic pathogens. The study will also investigate genetic relationships between bacteria isolated from fruits and vegetables originated from different countries in the world. Findings from this study will provide information on the sanitary conditions of fruits and vegetables produced and consumed in this part of the world, which will help in improving their quality and safety for consumption. To our knowledge, this is the first report that analyzes microbial content of fresh produce in this country and also relates the presence of particular opportunistic pathogens isolated from different local and imported samples to particular effects on human health.

## 2. Materials and Methods

### 2.1. Sample Collection

Fruits and vegetables that are mostly eaten raw were selected for this study. These contained 7 types of fresh imported or locally produced fruits (banana,* Musa* spp.; dates,* Phoenix dactylifera*; mango,* Mangifera indica*; papaya,* Carica papaya*; pomegranate,* Punica granatum*; tomato,* Solanum lycopersicum*; and watermelon,* Citrullus lanatus*) and 6 types of fresh imported or locally produced vegetables (cabbage,* Brassica oleracea*; capsicum,* Capsicum annuum*; carrot,* Daucus carota*; cucumber,* Cucumis sativus*; lettuce,* Lactuca sativa*; and radish,* Raphanus sativus*). Samples were purchased from local markets in Muscat or Nizwa, Oman, during the period of April to September 2014. The imported fruits and vegetables originated from different countries and they were selected depending on their availability in the market during that period of time. Three samples were obtained for each fruit or vegetable type originated from the same country. The samples were collected aseptically and refrigerated until analyzed within 24 hrs.  Table 1 represents the origin of all samples.

### 2.2. Microbiological Analysis of Fruits and Vegetables

#### 2.2.1. Sample Preparation, Aerobic Plate Count, and Selective Bacterial Counts

Samples were analyzed in a safety cabinet (Purifier class II, Labconco, Kansas, USA) and cut using sterile scalpels. Some fruits (banana, mango, papaya, pomegranate, and watermelon) were peeled and the inside flesh was analyzed. Twenty-five grams of the cut sample was weighed in a sterile stomacher bag and mixed with 225 mL of Maximum Recovery Diluent (MRD) and homogenized for 1 min using a stomacher (Bagmixer 100 MiniMix, Interscience, Bois Arpents, France). Serial dilutions were prepared from the original homogenate in MRD. Aerobic plate count (APC) was performed by spread plate method. The plates of standard plate count agar (SPCA) were incubated at 35°C for 48 hrs [7]. The count of Enterobacteriaceae was performed on Violet Red Bile Glucose (VRBG) agar by pour plate method and incubation at 35°C for 24 hrs.* E. coli* was counted on Tryptone Bile X-glucuronide (TBX) medium and the plates were incubated at 2 temperatures: 30 and 44°C for 24 hrs [14].* Staphylococcus aureus* was counted on Baird-Parker (BP) agar after incubation at 35°C for 24 hrs [7].* Enterococcus* was counted on Slanetz agar after incubation at 35°C for 48 hrs [15]. All microbiological media were from Oxoid, England, and all experiments were repeated three times.

#### 2.2.2. Identification of Bacteria

For each selective medium (BPA, VRBG, TBX, and Slanetz agar), one bacterial isolate showing the typical morphology (details could be found by searching manufacturer's website (http://www.oxoid.com/UK/blue/index.asp?c=UK&lang=EN)) was selected from each sample for identification. Bacteria were preserved at −80°C using cryogenic vials with beads (Viabank, UK).

*(1) Identification of Bacteria by VITEK 2*. Identification of bacteria using biochemical tests was done using an automated identification equipment; VITEK 2-compact 15 (bioMérieux, France) following instructions of the manufacturer. In brief, bacterial suspensions were prepared in sterile saline (0.45%) and the density was adjusted to a McFarland standard of 0.5–0.63 using VITEK 2 DensiCheck (bioMérieux, France). GP and GN cards were used for Gram-positive and Gram-negative bacteria, respectively.

*(2) Identification of Bacteria by Polymerase Chain Reaction (PCR)*. The second method was performed using polymerase chain reaction (PCR) targeting bacterial 16S rRNA [16]. Briefly, DNA was extracted using “foodproof StarPrep Two Kit” (Biotecon Diagnostics GmbH, Potsdam, Germany). The quality and quantity of DNA were checked using NanoDrop*™* 2000 (Thermoscientific, USA). PCR was done by transferring 1 *μ*L of each primer (27F and 1492R; DNA sequences: 5′-AGAGTTTGATCMTGGCTCAG-3′ and 5′-TACGGYTACCTTGTTACGACTT-3′, resp.), 22 *μ*L of sterile milliQ water, and 1 *μ*L of the DNA sample to PCR reaction tubes containing PCR beads (puReTaq Ready-To-Go PCR beads, GE Healthcare, UK). The thermal profile (Veriti 96-well Thermal cycler, Applied Biosystems, Singapore) for PCR reaction was as follows: denaturation at 95°C for 2 min, followed by 35 cycles of denaturation at 95°C for 30 sec, annealing at 54°C for 30 sec, and extension at 72°C for 1 min. The final extension was at 72°C for 10 min and then kept at 4°C. Aliquots of PCR products were checked by subjecting them to 1.5% agarose gel electrophoresis and viewing them by GelDoc. PCR products were sent for sequencing at Macrogen (Korea) using the same primers used for amplification. The sequencing results were compared with those found at the National Center for Biotechnology Information (NCBI) using BLAST search. Then, sequences of reference isolates of each bacterial species were obtained from GenBank and neighbor joining trees were constructed for each bacterial group/genus using the Kimura 2 parameter evolutionary model (Mega 5) [17]. Then bootstrap 50% majority-rule consensus trees were generated (1000 replications).

### 2.3. Statistical Methods

Two-way analysis of variance (ANOVA) was used to determine if aerobic plate counts and Enterobacteriaceae counts varied significantly between different types of fruits/vegetables and between local and imported fruits/vegetables. Data Disk 6.1 (Data Description, Inc., New York, USA) was used to perform the statistical tests to identify any significant differences which were considered as *P* < 0.05.

## 3. Results

### 3.1. Bacterial Counts

**Figure 1:**
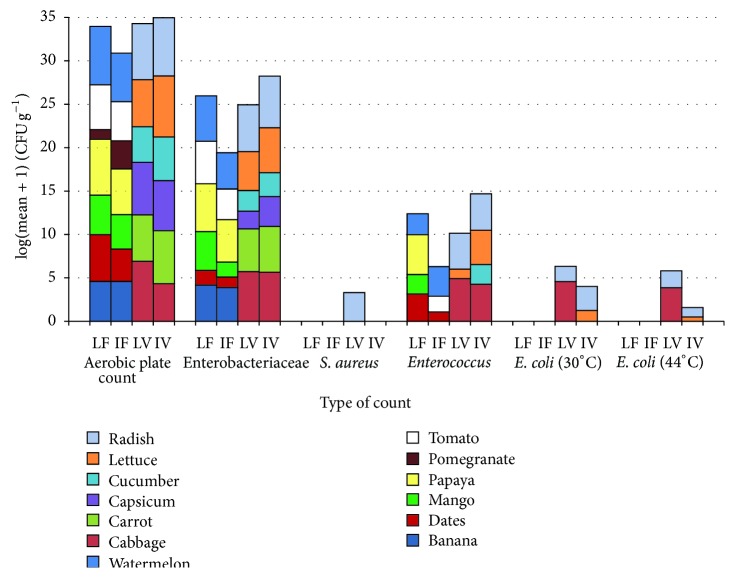
Microbial counts (log (mean + 1) CFU g^−1^) in local fruits (LF), imported fruits (IF), local vegetables (LV), and imported vegetables (IV).

The origin and number of positive samples of fruits and vegetables are shown in Table 1 while Figure 1 shows the counts of different bacterial groups in different fruits and vegetables used in this study. Aerobic plate count (APC) of local fruits (LF) (mean = 6.1 log CFU g^−1^) was significantly greater than APC of imported fruits (IF) (mean = 5.0 log CFU g^−1^) (ANOVA, *P* = 0.0227, *α* = 0.05) but APC was not significantly different between local vegetables (LV) (mean = 6.3 log CFU g^−1^) and imported vegetables (IV) (mean = 6.5 log CFU g^−1^) (ANOVA, *P* = 0.2046, *α* = 0.05). APC differed significantly between the different types of fruits (ANOVA, *P* = 0.0001, *α* = 0.05) and vegetables (ANOVA, *P* = 0.0035, *α* = 0.05). Enterobacteriaceae and* Enterococcus* were found in all groups that were tested, LF, IF, LV, and IV while* S. aureus* was found only in LV (radish).* E. coli* was isolated from LV and IV but not from any fruit (Figure 1). Fifteen samples (71.4%, *n* = 21) of the locally produced fruits were positive for Enterobacteriaceae (mean = 4.9 log CFU g^−1^) while about half (53.8%, *n* = 39) of the imported fruits were positive for Enterobacteriaceae (mean = 4.2 log CFU g^−1^). Seventeen samples (94.4%, *n* = 18) of the local vegetables were positive for Enterobacteriaceae (mean = 5.2 log CFU g^−1^) while 24 samples (88.9%, *n* = 27) of the imported vegetables were positive (mean = 5.3 log CFU g^−1^). The count of Enterobacteriaceae in LF was significantly greater than the count in IF (ANOVA, *P* = 0.0011, *α* = 0.05) but it did not differ significantly between LV and IV (ANOVA, *P* = 0.2029, *α* = 0.05). The count of Enterobacteriaceae was significantly different from one type of fruit to another (ANOVA, *P* = 0.0001, *α* = 0.05) as well as among different types of vegetables (ANOVA, *P* = 0.0001, *α* = 0.05).

At 30°C,* E. coli* was isolated from 6 LV (3 cabbages and 3 radishes, mean = 3.8 log CFU g^−1^) and from 2 IV samples (lettuce and radish, mean = 1.9 log CFU g^−1^) while at 44°C* E. coli* was isolated from 4 LV samples (3 cabbages and 1 radish, mean = 3.1 log CFU g^−1^) and from 3 IV samples (2 lettuces and 1 radish, mean = 0.4 log CFU g^−1^).* Enterococcus* was found in 6 LF (28.6%, mean = 3.8 log CFU g^−1^), 6 IF (15.4%, mean = 2.6 log CFU g^−1^), 7 LV (38.9%, mean = 4.2 log CFU g^−1^), and 13 IV (48.1%, mean = 3.8 log CFU g^−1^).* S. aureus* was isolated only from 3 local radish samples (mean = 2.5 log CFU g^−1^).

### 3.2. Identification of Bacteria

Out of 130 bacteria (numbered from 1–130) that were isolated from fresh fruits and vegetables (except 4 reference strains, numbers 127–130), a total of 97 (74.6%) isolates (21 species) were similarly identified by VITEK 2 and PCR to species level (Table 2). The most encountered species was* E. coli* (15 isolates) followed by* Klebsiella pneumoniae* and* Enterococcus casseliflavus* (13 isolates each) and then* Enterobacter cloacae* (12 isolates). Thirty-three bacteria were not similarly identified to species level by VITEK 2 and PCR (Table 3). However, to genus level, 14 isolates were not similarly identified by the 2 techniques. The differences in identification at genus level were only among isolates of Enterobacteriaceae. The 16S rRNA gene sequences of bacteria numbers 1–130 were sequentially given accession numbers of KR265345-KR265474 by the GenBank.

### 3.3. Phylogenetic Analysis

**Figure 2:**
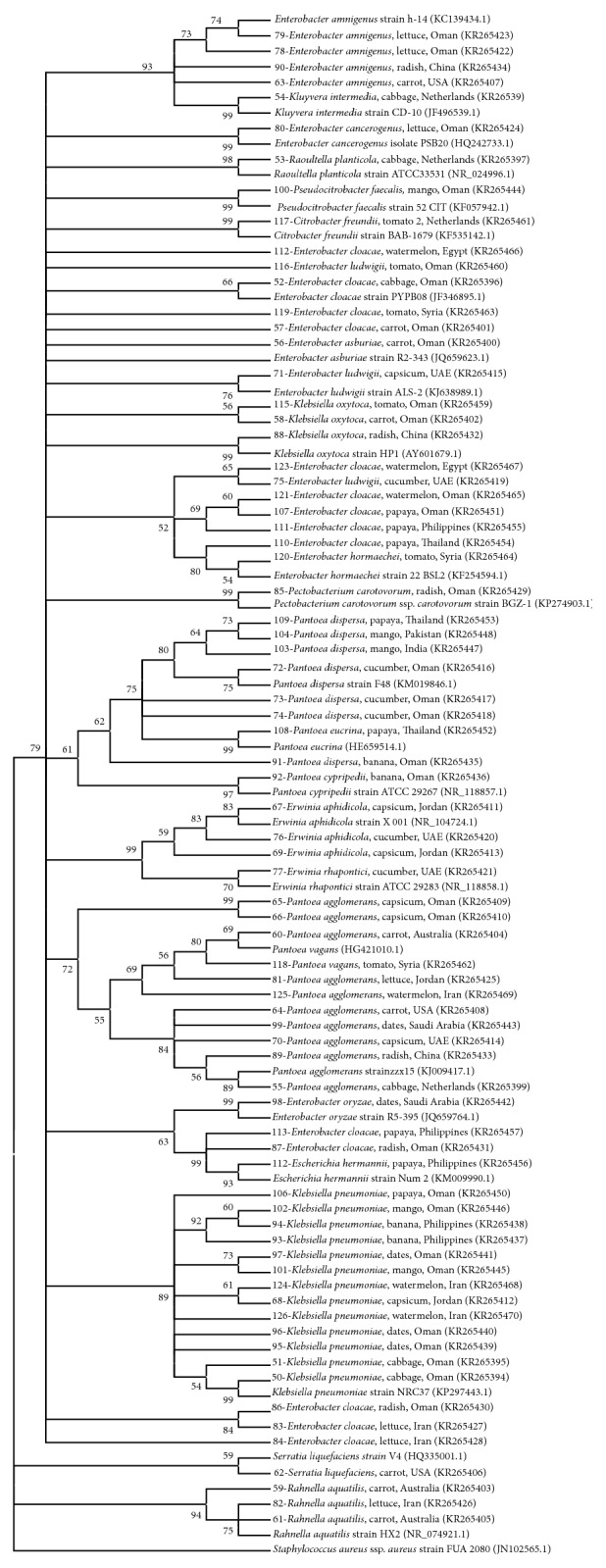
Neighbor joining tree based on 16S rRNA gene sequences of members of Enterobacteriaceae isolated from fresh produce.* Staphylococcus aureus* (JN102565) was included as an outgroup. KR-accession numbers correspond to gene sequences that belong to isolates analyzed in this study while others were obtained from NCBI database. Bootstrap values above 50% are shown (1000 replications).

**Figure 3:**
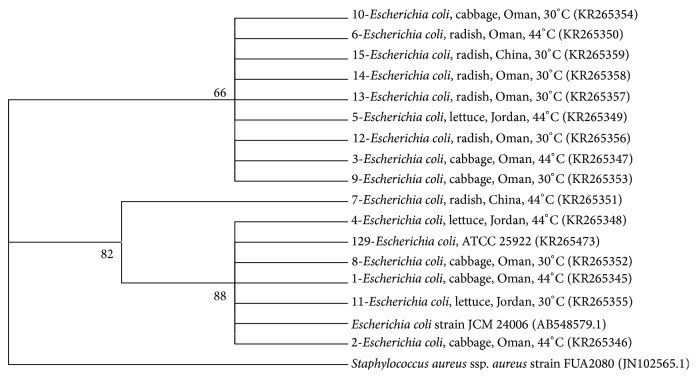
Neighbor joining tree based on 16S rRNA gene sequences of* E. coli* isolated from fresh produce.* E. coli* strain JCM 24006 (AB548579) was included as a reference strain and* Staphylococcus aureus* (JN102565) was included as an outgroup. KR-accession numbers correspond to gene sequences that belong to isolates analyzed in this study while AB548579 and JN102565 were obtained from the NCBI database. Bootstrap values above 50% are shown (1000 replications).

**Figure 4:**
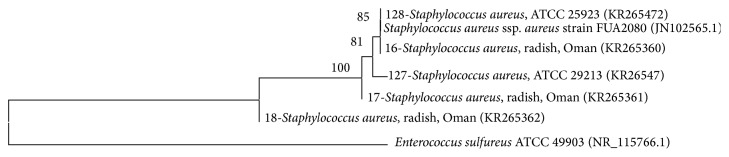
Neighbor joining tree based on 16S rRNA gene sequences of* Staphylococcus aureus* isolated from local radish.* Enterococcus sulfureus* ATCC 49903 (NR115766) was included as an outgroup. KR-accession numbers correspond to gene sequences that belong to isolates analyzed in this study while others were obtained from NCBI database. Bootstrap values above 50% are shown (1000 replications).

**Figure 5:**
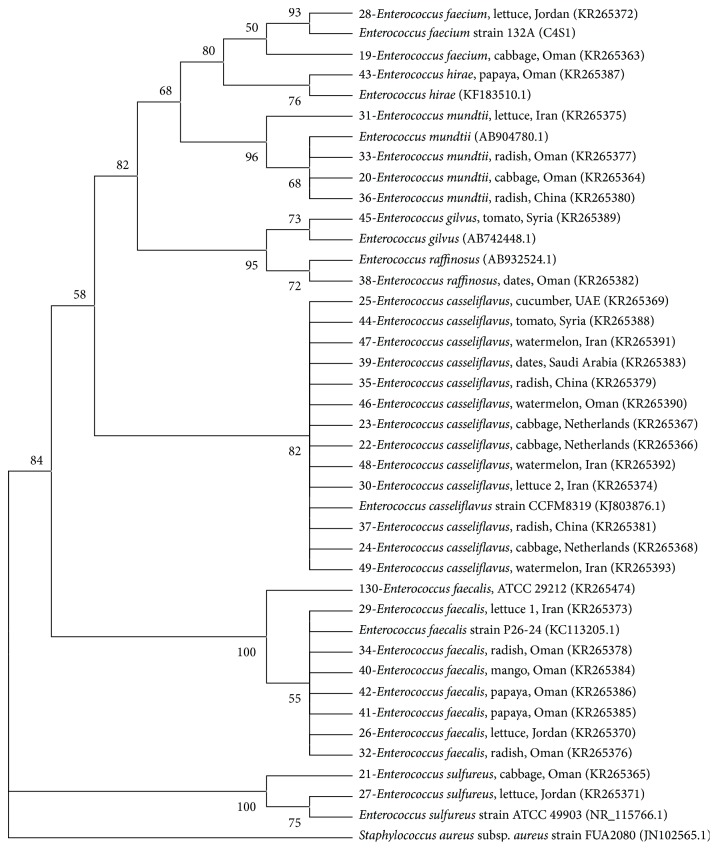
Neighbor joining tree based on sequencing 16S rRNA gene for members of* Enterococcus* isolated from fresh produce.* Staphylococcus aureus* (JN102565) was included as an outgroup. KR-accession numbers correspond to gene sequences that belong to isolates analyzed in this study while others were obtained from NCBI database. Bootstrap values above 50% are shown (1000 replications).

Phylogenetic analysis showed grouping of most bacterial isolates with the reference species obtained from NCBI, except for few isolates from the Enterobacteriaceae.* K. pneumoniae* and* R. aquatilis* made distinct clear clusters while other isolates belonging to Enterobacteriaceae made several groups (Figure 2).* E. coli* isolates grouped into two clusters, (bootstrap values: 66–86%; Figure 3). However, there was no relationship between clustering of isolates and the countries or commodities from which they were obtained. The 5 isolates (including 2 reference strains) of* S. aureus* also grouped together with a very high bootstrap support (100%; Figure 4).* Enterococcus* species grouped into different clusters (50–100% bootstrap support; Figure 5). There was also no relationship between clustering of isolates of the* Enterococcus* species and the countries or commodities from which they were obtained (Figure 5).

## 4. Discussion

Most studies report the presence of pathogens in fruits and vegetables but the counts are rarely documented [5]. APC can be used to monitor the hygienic quality of a product throughout processing and distribution [18] but it is difficult to be used to draw a conclusion on the safety of a product [19]. Nevertheless, some researchers [20] found a strong association between APC and presence of* E. coli* in beef carcasses where 88% of samples having APC of ≥4 log CFU/cm^2^ were positive for* E. coli* while only 21% of samples having APC of <2 log CFU/cm^2^ were positive. In this study,* E. coli* was isolated only from vegetable samples (from local cabbage and radish and from imported lettuce and radish) all with mean APC ≥ 6.5 log CFU g^−1^ (Figure 1). It is possible that a certain association does exist between APC and a particular pathogen but it depends on the type of a product and needs a large number of samples to be tested.

In the current study, vegetables showed slightly larger APCs than fruits (range of mean count = 4.1–7.0 and 1.1–6.7 log CFU g^−1^, resp.). Vegetables are usually grown closer to soil than fruits and thus might be contaminated easily from soil [21]. In addition, some fruits that were observed to be physically contaminated with soil (watermelon) or deposited with dust (papaya) showed high APC counts of ≥6 log CFU g^−1^. Leafy vegetables were reported to give greater APC than nonleafy vegetables [7]. The highest microbial counts were obtained from the APC of leafy vegetables, imported lettuce (mean = 7.0 log CFU g^−1^) followed by local cabbage (mean = 6.9 log CFU g^−1^). This is 1 log less than the maximum APC (8 log CFU g^−1^) that was reported in cantaloupe, spinach, and lettuce in Saudi Arabia [7] but their maximum Enterobacteriaceae count of 4 log CFU g^−1^ (cabbage and lettuce) was about 2 logs less than what was found in the current study (papaya, cabbage, and radish) (Figure 1). Pomegranate was found to be the least contaminated fruit having APC of 1.1 and 3.2 log CFU g^−1^ (local and imported, resp.) with none of the other sought bacterial groups being detected. Pomegranate juice [2] and extracts [22, 23] were shown to possess antibacterial activity but the low pH of about 3.5 of pomegranate [24] might also have contributed to this inhibitory activity.

Clinically, many members of the family of Enterobacteriaceae are among the most potent and prevalent pathogens [25]; indeed, many of them have acquired resistance to most antibiotics [21]. Once ingested, antibiotic resistance genes, if present, may be transferred to the normal flora of the human gut and thus possibly to the pathogenic bacteria [26] especially if these resistance determinants can persist in the human gut for as long as a year as was proposed by Forslund and colleagues [27]. Many outbreaks in association with consumption of fresh produce are due to bacteria belonging to Enterobacteriaceae. Outbreaks that were linked to* Salmonella* Poona in cucumbers,* S.* Enteritidis in bean sprouts,* E. coli* O121 in raw clover sprouts, in 2014, and* E. coli* O157:H7 in ready-to-eat salads in 2013 are just some examples [10]. The normal flora of many fruits and vegetables contains bacteria within the group of Enterobacteriaceae that might be present at high counts [28]. Our results showed that the counts of Enterobacteriaceae were comparable to APCs (Figure 1) and only pomegranate was free from bacteria belonging to this family (Table 1). A total of 15 species of Enterobacteriaceae were similarly identified by VITEK 2 and PCR (Table 2). In addition, 11 other species within this group were identified by PCR but not by VITEK 2 (Table 3). The 3 genera of* Erwinia*,* Pectobacterium,* and* Pseudocitrobacter* are not included in the most updated version (7.01) of the VITEK 2 identification database by GN card (isolates numbers 67, 69, 76, 77, 85, and 100) and 6 other isolates could have been misidentified at genus level by VITEK 2 as compared to PCR (isolates numbers 80, 83, 87, 91, 112, and 113) though these genera are included in VITEK 2 database. Within this group, 5 species of the similarly identified genera by VITEK 2 and PCR are not included in VITEK 2 latest version database including* Pantoea dispersa* (isolates numbers 72, 73, 74, 103, 104, and 109),* Pantoea cypripedii* (isolate number 92),* Enterobacter oryzae* (isolate number 98),* Pantoea eucrina* (isolate number 108), and* Pantoea vagans* (isolate number 118).

The genus of* Klebsiella* harbors opportunistic pathogens capable of causing severe infections [29].* Klebsiella pneumoniae* was one of the most dominant species in this study and was isolated from local cabbage, dates, mango, and papaya and from imported capsicum (Jordan), banana (Philippine), and watermelon (Iran).* Pantoea agglomerans* (formerly* Enterobacter agglomerans*) and* Rahnella aquatilis* are opportunistic pathogens as well [30]. The genus of* Enterobacter* is one of the largest genera in the family Enterobacteriaceae having at least 19 species. It also shows polyphyletic patterns when phylogenetic trees are constructed based on 16S rRNA sequences [31]. Many species within the genus of* Enterobacter* have been marked as emerging pathogens [30].* Pseudocitrobacter* is a new genus in the family of Enterobacteriaceae and* Pseudocitrobacter faecalis* was isolated from a stool sample of a patient and was found to possess NDM-1 carbapenemase which confers resistance to carbapenem antibiotics [32]. In this study, one isolate was identified as* P. faecalis* (from local mango) by PCR (Table 3). To our knowledge, this is the first report of isolation of this species from fresh produce.

*Erwinia aphidicola* was first isolated from the gut of pea aphid by Harada and coworkers [33]. In their study, biochemical tests showed that this species was the closest to* Erwinia herbicola* and* Pantoea agglomerans* but DNA-DNA hybridization separated it into a new species. Later on, this species was recognized as an important plant pathogen especially for beans [34]. In this study, the 3 isolates of* E. aphidicola* (from imported capsicum and cucumber) as identified by PCR were identified as* Pantoea* spp. by VITEK 2 and this could be because of their similarities based on biochemical reactions.* Rahnella aquatilis*, though is commonly present in vegetables [28], has been found to be a primary pathogen and was isolated from blood, sputum, urine, and stool [35]. In this study,* R. aquatilis* was isolated from carrot (Australia) and lettuce (Iran) (Table 2). Likewise,* Serratia* spp. are often present in vegetables [28] but a species such as* Serratia marcescens* (isolated from local tomato in this study) has been recognized as an important cause of nosocomial infections [36].* Serratia liquefaciens* was also found to cause bloodstream infection [37].* Citrobacter freundii*, isolated from tomato, Netherlands, in this study, was reported to cause nosocomial bacteremia [38].* Raoultella planticola *(isolated from cabbage, Netherlands) was found to cause wound infection [39].

*E. coli* is considered a better sanitary indicator than Enterobacteriaceae [28] where its presence indicates recent fecal contamination [30]. Unlike Österblad and colleagues [40] who rarely found* E. coli* in the examined 137 vegetable samples,* E. coli* was the most encountered species in this study properly due to its selective isolation on TBX medium. According to Public Health Leadership Society [41], fresh produce with* E. coli* count of ≥2 log CFU g^−1^ is considered unsatisfactory. In this study, local cabbage and radish and imported radish contained this unsatisfactory level (Figure 1). In the current study, prevalence of* E. coli* in local and imported vegetables (33%, *n* = 18 and 15%, *n* = 27, resp., or 22% in all vegetables) is greater than what was found by Abadias and coworkers [28] in whole vegetables (7%, *n* = 28). The count of* E. coli* isolated at 44°C was less than the count at 30°C (Figure 1) indicating that, for direct counting of* E. coli* at 44°C on TBX, it would be better to preincubate plates at 30°C in order to resuscitate any injured cells.

*S. aureus* was found only in local radish with a mean count of 2.5 log CFU g^−1^. It was reported that high levels of* S. aureus* of >4 log CFU g^−1^ might indicate enterotoxin production [14]. However, other researchers [42] reported that* S. aureus* was attributed to be the cause of a food poisoning outbreak in a hotel in Japan in 2005 where pickled radish was one of the sources that* S. aureus* was isolated from. The counts of this pathogen in radish, sashimi, and frozen crab were 1.7, 1.7, and 2.0 log CFU g^−1^, respectively, but its enterotoxin was found in a vomit sample indicating that isolation of pathogens from foods is significant, even at low levels, as some of them might proliferate in the food products if they are not kept at proper temperature (1–5°C for fresh fruits and vegetables) [28].

Similar to previous findings [43], our results showed that more vegetable samples (LV = 38.9%, IV = 48.1%) harbor* Enterococcus* than fruits (LF = 28.6%, IF = 15.4%).* Enterococcus* spp. are involved in food intoxication and in spreading antibiotic resistance through the food chain and they are a leading cause of nosocomial infections [15] where* E. faecalis* and* E. faecium* cause the majority of them [43].* Enterococcus* was isolated from all types of the tested fruits and vegetables (local and/or imported) except banana, pomegranate, carrot, and capsicum (Table 1). The highest counts of* Enterococcus* were found in local cabbage followed by local papaya (4.9 and 4.6 log CFU g^−1^, resp., Figure 1). The presence of* Enterococcus* indicates fecal contamination [30] that might not be recent as they can persist for long time in the environment. However, besides gastrointestinal tract of animals and humans, this ubiquitous organism can be found in soil and on plants but identification of species might help in discriminating the source of* Enterococcus*.* E. faecalis* and* E. faecium* might be a better indicator of human fecal contamination than* Enterococcus casseliflavus* and* Enterococcus mundtii* that are more prevalent in environment [44]. In this study,* E. faecalis* was isolated from lettuce imported from Jordan and Iran and from radish, mango, and papaya produced in Oman while* E. faecium* was isolated from cabbage produced in Oman and from lettuce imported from Jordan. In other studies,* E. faecalis* was isolated from lettuce, radish, tomato, apple, cucumber, potato, sprouts, and carrot while* E. faecium* was isolated from dates, lettuce, tomato, and soy product [15, 43].

Similar to the result of others [43],* E. casseliflavus* was the most common species of* Enterococcus* (13 isolates, Table 2). Three isolates of* Enterococcus ludwigii* (capsicum, cucumber (UAE), tomato (Oman)), 1 isolate of* Enterococcus hirae* (papaya, Oman), and 1 isolate of* Enterococcus raffinosus* (dates, Oman) were found. Three species within the genus of* Enterococcus* that were not similarly identified by VITEK 2 and PCR are not included in the VITEK 2 latest version database including* E. mundtii* (isolates numbers 20, 31, 33, and 36),* E. sulfureus* (isolates numbers 21 and 27), and* E. gilvus* (isolate number 45).* E. hirae* and* E. mundtii* were isolated previously from different fruits and vegetables [43]. It was shown that genetic exchange of virulence determinants can occur between food and clinical isolates of* E. faecium* [45]. In fact, contaminated food might pose a risk and be a source for nosocomial infections [46].

Table 4 summarizes some bacteria that were previously reported to cause infections in human and that were isolated in the present study from fresh produce. Their sources could be environmental, food, or unknown. Clearly, these bacteria can gain access to critical sites in the body and establish infections. Many of them are considered as opportunistic pathogens capable of establishing infections in immunocompromised individuals. However, there are some sporadic cases where they can act as primary pathogens and thus cause infections in immunocompetent healthy people. Moreover, it is possible that opportunistic pathogens might emerge as important pathogens in future [3]. Determining the microbial quality of fresh produce served in hospitals would be of particular importance because translocation of pathogenic or opportunistic pathogens either to immunocompromised patients or to other environments in the hospitals might give these bacteria and/or their pathogenicity genetic determinants a better chance to be involved in the disease process.

Phylogenetic analysis showed grouping of most bacterial isolates with the reference strains obtained from NCBI, except for few species mainly from the Enterobacteriaceae. Members within Enterobacteriaceae have close genetic relationships and species often give polyphyletic groups in trees constructed using 16S rRNA sequences [63–65]. The 5 isolates of* S. aureus* were grouped together with a very high bootstrap support (100%, Figure 4). Likewise, it was shown that sequencing of 16S rRNA gene could be accurately used to generate phylogenetic relationships of the genus* Staphylococcus* [66]. Sequencing of 16S rRNA gene gave good discrimination of the genus* Enterococcus* into 8 clusters, namely,* E. faecium*,* E. hirae*,* E. mundtii*,* E. gilvus*,* E. raffinosus*,* E. casseliflavus*,* E. faecalis*, and* E. sulfureus* (Figure 5, bootstrap percentages 50–100). Similar discrimination of the genus* Enterococcus* was previously shown by sequencing 16S rRNA and* rpoA* genes [67].

Although bacteria can contaminate fresh produce at any stage from farm to fork, in the current study, some samples were peeled and many were obtained in their original packages which may suggest contamination from the original countries. No relationship was found between clustering of the isolates based on the 16S rRNA gene and the countries or commodities from which they were obtained. Thus, circulation of these bacterial species among different countries cannot be ruled out and this may raise concerns about the role played by quarantine in limiting spread of human and plant pathogens via agricultural commodities. However, more epidemiological studies should be done specifically for each species taking into account increasing the number of isolates and further characterizing the strains in order to establish some relationships between the different genotypes.

In conclusion, this study analyzed for the first time the microbial load of some fresh fruits and vegetables in Oman at the point of retail sale. The presence of high counts of APC and Enterobacteriaceae as well as fecal bacteria indicates that the hygienic conditions of fruits and vegetables in Oman are not satisfactory and should be improved. Understanding the complex microbial ecosystem that is unique for each produce in a given country would be important to establish fully controlled systems for fruits and vegetables that can be applied during harvest, production, distribution, and marketing aimed at controlling spoilage and pathogenic microbes. The presence of* S. aureus*, possible pathogenic enterococci, and* E. coli* as well as other opportunistic and emerging pathogens in the examined fresh fruits and vegetables requires further investigation of virulence determinants which might be important in future for tracking their pathogenicity evolution.

## Figures and Tables

**Table 1:** Origin of fruits and vegetables and the number of positive samples for different microbial counts.

Number	Type of produce	Origin^*∗*^	APC	Enterobacteriaceae	*S. aureus*	*Enterococcus*	*E. coli* (30°C)	*E. coli* (44°C)
	Fruit (*n* = 60)							
1	Banana	Oman	3	3	0	0	0	0
		Philippine	3	2	0	0	0	0
2	Dates	Oman	3	3	0	1	0	0
		Saudi Arabia	3	2	0	1	0	0
3	Mango	Oman	3	3	0	1	0	0
		India, Pakistan	3, 3	1, 1	0, 0	0, 0	0, 0	0, 0
4	Papaya	Oman	3	3	0	3	0	0
		Thailand, Philippine	3, 3	3, 3	0, 0	0, 0	0, 0	0, 0
5	Pomegranate	Oman	1	0	0	0	0	0
		India, Saudi Arabia	2, 3	0, 0	0, 0	0, 0	0, 0	0, 0
6	Tomato	Oman	3	3	0	0	0	0
		Jordan, Netherlands, Syria	2, 1, 3	0, 1, 3	0, 0, 0	0, 0, 2	0, 0, 0	0, 0, 0
7	Watermelon	Oman	3	1	0	1	0	0
		Egypt, Iran	3, 3	2, 3	0, 0	0, 3	0, 0	0, 0

	Vegetable (*n* = 45)							
1	Cabbage	Oman	3	3	0	3	3	3
		Netherlands	3	3	0	3	0	0
2	Carrot	Oman	3	3	0	0	0	0
		Australia, USA	3, 3	3, 3	0, 0	0, 0	0, 0	0, 0
3	Capsicum	Oman	3	2	0	0	0	0
		Jordan, UAE	3, 3	3, 2	0, 0	0, 0	0, 0	0, 0
4	Cucumber	Oman	3	3	0	0	0	0
		UAE	3	3	0	1	0	0
5	Lettuce	Oman	3	3	0	1	0	0
		Iran, Jordan	3, 3	3, 1	0, 0	3, 3	0, 1	0, 2
6	Radish	Oman	3	3	3	3	3	1
		China	3	3	0	3	1	1

APC: aerobic plate count, USA: United States of America, and UAE: United Arab Emirates.

^*∗*^Three samples were analyzed from each country.

**Table 2:** Bacteria that were similarly identified to species level by VITEK 2 and PCR and their sources.

Name of bacteria	Number of isolates	Rank	Source
*Citrobacter freundii *	1	13	Tomato (Netherlands)
*Enterobacter amnigenus*	4	7	Carrot (USA), lettuce (Oman), radish (China)
*Enterobacter asburiae*	1	13	Carrot (Oman)
*Enterobacter cloacae*	12	4	Cabbage, carrot, papaya, radish, watermelon (Oman), lettuce (Iran), papaya (Philippines), papaya (Thailand), tomato (Syria), watermelon (Egypt)
*Enterobacter hormaechei *	1	13	Tomato (Syria)
*Enterobacter ludwigii *	3	8	Capsicum, cucumber (UAE), tomato (Oman)
*Enterococcus casseliflavus*	13	2	Cabbage (Netherlands), cucumber (UAE), dates (Saudi Arabia), lettuce (Iran), radish (China), tomato ( Syria), watermelon (Iran), watermelon (Oman)
*Enterococcus faecalis*	7	5	Lettuce (Jordan), lettuce (Iran), radish, mango, papaya (Oman)
*Enterococcus faecium*	2	11	Cabbage (Oman), lettuce (Jordan)
*Enterococcus hirae*	1	13	Papaya (Oman)
*Enterococcus raffinosus*	1	13	Dates (Oman)
*Escherichia coli*	15	1	Cabbage, radish (Oman), lettuce (Jordan), radish (China)
*Klebsiella oxytoca*	2	11	Radish (China), tomato (Oman)
*Klebsiella pneumoniae*	13	2	Cabbage (Oman), capsicum (Jordan), banana (Philippines), dates (Oman), mango (Oman), papaya (Oman), watermelon (Iran)
*Kluyvera intermedia *	1	13	Cabbage (Netherlands)
*Pantoea agglomerans*	7	5	Cabbage (Netherlands), carrot (Australia), carrot (USA), capsicum (UAE), lettuce (Jordan), radish (China), watermelon (Iran)
*Rahnella aquatilis*	3	8	Carrot (Australia), lettuce (Iran)
*Raoultella planticola*	1	13	Cabbage (Netherlands)
*Serratia liquefaciens*	1	13	Carrot (USA)
*Serratia marcescens*	1	13	Tomato (Oman)
*Staphylococcus aureus*	3	8	Radish (Oman)

Reference strains			
*Staphylococcus aureus*	1		ATCC 29213
*Staphylococcus aureus*	1		ATCC 25923
*Escherichia coli*	1		ATCC 25922
*Enterococcus faecalis*	1		ATCC 29212
Total	97		

**Table 3:** Bacteria that were not similarly identified to species level by VITEK and PCR and their sources.

Number of strains	VITEK	PCR	Source
20	*Enterococcus faecium *or *E. gallinarum* ^*∗*^	*Enterococcus mundtii*	Cabbage (Oman)
21	*Enterococcus cecorum *or *Leuconostoc pseudomesenteroides* ^*∗*^	*Enterococcus sulfureus *	Cabbage (Oman)
27	*Enterococcus cecorum* or *Leuconostoc pseudomesenteroides* ^*∗*^	*Enterococcus sulfureus*	Lettuce (Jordan)
31	*Enterococcus faecium* or *E. gallinarum* ^*∗*^	*Enterococcus mundtii *	Lettuce (Iran)
33	*Enterococcus faecium* or *E. gallinarum* ^*∗*^	*Enterococcus mundtii*	Radish (Oman)
36	*Enterococcus faecium* or *E. gallinarum* ^*∗*^	*Enterococcus mundtii*	Radish (China)
45	*Enterococcus raffinosus*	*Enterococcus gilvus*	Tomato (Syria)
58	*Klebsiella pneumoniae* ssp. *pneumoniae*	*Klebsiella oxytoca*	Carrot (Oman)
65	*Pantoea *spp. or *Aeromonas sobria* ^*∗*^	*Pantoea agglomerans*	Capsicum (Oman)
66	*Pantoea *spp.	*Pantoea agglomerans*	Capsicum (Oman)
67	*Pantoea* spp.	*Erwinia aphidicola *	Capsicum (Jordan)
69	*Pantoea* spp.	*Erwinia aphidicola*	Capsicum (Jordan)
72	*Ewingella americana* or *Pantoea *spp.^*∗*^	*Pantoea dispersa*	Cucumber (Oman)
73	*Pantoea* spp.	*Pantoea dispersa*	Cucumber (Oman)
74	*Pantoea* spp.	*Pantoea dispersa*	Cucumber (Oman)
76	*Pantoea* spp.	*Erwinia aphidicola*	Cucumber (UAE)
77	*Pantoea* spp.	*Erwinia rhapontici*	Cucumber (UAE)
80	*Leclercia adecarboxylata*	*Enterobacter cancerogenus*	Lettuce (Oman)
83	*Pantoea* spp.	*Enterobacter cloacae*	Lettuce (Iran)
85	*Pantoea* spp.	*Pectobacterium carotovorum*	Radish (Oman)
87	*Pantoea* spp.	*Enterobacter cloacae *	Radish (Oman)
91	*Serratia ficaria *	*Pantoea dispersa*	Banana (Oman)
92	*Pantoea* spp.	*Pantoea cypripedii*	Banana (Oman)
98	*Enterobacter cloacae* complex^*∗∗*^	*Enterobacter oryzae *	Dates (Saudi Arabia)
99	*Pantoea* spp.	*Pantoea agglomerans *	Dates (Saudi Arabia)
100	*Pantoea* spp.	*Pseudocitrobacter faecalis *	Mango (Oman)
103	*Pantoea* spp.	*Pantoea dispersa *	Mango (India)
104	*Pantoea *spp.	*Pantoea dispersa *	Mango (Pakistan)
108	*Pantoea* spp.	*Pantoea eucrina *	Papaya (Thailand)
109	*Pantoea* spp.	*Pantoea dispersa *	Papaya (Thailand)
112	*Pantoea *spp.	*Escherichia hermannii*	Papaya (Philippines)
113	*Pantoea* spp.	*Enterobacter cloacae *	Papaya (Philippines)
118	*Pantoea* spp.	*Pantoea vagans *	Tomato (Syria)

^*∗*^Low discrimination organisms (same biopattern was produced by the tested strains). ^*∗∗*^Slash line: biopattern is the same for these organisms: *Enterobacter kobei, E. hormaechei*, *E. cloacae* ssp. *cloacae*, *E. cloacae* ssp. *dissolvens,* or *E. ludwigii*.

**Table 4:** Case reports for bacterial infections caused by bacteria listed in Table 2 as presented in the literature.

Bacteria	Specimen of isolation	Country	Ref.
*Citrobacter freundii *	Blood, urine	USA, South Africa	[38, 47]
*Enterobacter amnigenus*	Eye	USA	[48]
*Enterobacter asburiae*	Blood	South Africa	[47]
*Enterobacter cloacae*	Stool	USA	[49]
*Enterobacter hormaechei *	Blood/trachea, nasopharynx/rectum	USA	[50]
*Enterobacter ludwigii *	Bone	India	[51]
*Enterococcus casseliflavus*	Cerebrospinal fluid	Italy	[52]
*Enterococcus faecalis*	Blood	South Africa	[47]
*Enterococcus faecium*	Cerebrospinal fluid	USA	[53]
*Enterococcus hirae*	Blood	France	[54]
*Enterococcus raffinosus*	Pelvic hematoma	Germany	[55]
*Escherichia coli*	Stool	USA	[49]
*Klebsiella oxytoca*	Stool	Austria	[56]
*Klebsiella pneumoniae*	Urine/liver, trachea	USA, South Africa	[47, 57]
*Pantoea agglomerans*	Blood	Italy	[58]
*Rahnella aquatilis*	Blood	Italy	[58]
*Raoultella planticola*	Peritoneal fluid, soft tissue, wound	Brazil, Ireland, Saudi Arabia	[39, 59, 60]
*Serratia liquefaciens*	Blood	USA	[37]
*Serratia marcescens*	Eye	USA	[61]
*Staphylococcus aureus*	Blood	Italy	[62]

## References

[1] Prasanna V., Prabha T. N., Tharanathan R. N. (2007). Fruit ripening phenomena—an overview. *Critical Reviews in Food Science and Nutrition*.

[2] Lee Y.-L., Cesario T., Wang Y., Shanbrom E., Thrupp L. (2003). Antibacterial activity of vegetables and juices. *Nutrition*.

[3] Berg G., Erlacher A., Smalla K., Krause R. (2014). Vegetable microbiomes: is there a connection among opportunistic infections, human health and our 'gut feeling'?. *Microbial Biotechnology*.

[4] Lozupone C. A., Stombaugh J. I., Gordon J. I., Jansson J. K., Knight R. (2012). Diversity, stability and resilience of the human gut microbiota. *Nature*.

[5] Olaimat A. N., Holley R. A. (2012). Factors influencing the microbial safety of fresh produce: a review. *Food Microbiology*.

[6] Beuchat L. R. (2002). Ecological factors influencing survival and growth of human pathogens on raw fruits and vegetables. *Microbes and Infection*.

[7] Hassan S. A., Altalhi A. D., Gherbawy Y. A., El-Deeb B. A. (2011). Bacterial load of fresh vegetables and their resistance to the currently used antibiotics in Saudi Arabia. *Foodborne Pathogens and Disease*.

[8] Al-Abri S. S., Al-Jardani A. K., Al-Hosni M. S., Kurup P. J., Al-Busaidi S., Beeching N. J. (2011). A hospital acquired outbreak of *Bacillus cereus* gastroenteritis, Oman. *Journal of Infection and Public Health*.

[9] Hauswaldt S., Nitschke M., Sayk F., Solbach W., Knobloch J. K.-M. (2013). Lessons learned from outbreaks of Shiga toxin producing *Escherichia coli*. *Current Infectious Disease Reports*.

[10] Centers for Disease Control and Prevention (CDC) http://www.cdc.gov/outbreaks/.

[11] Leff J. W., Fierer N. (2013). Bacterial communities associated with the surfaces of fresh fruits and vegetables. *PLoS ONE*.

[12] Opara L. U., Al-Said F. A., Al-Abri A. (2007). Assessment of what the consumer values in fresh fruit quality: case study of Oman. *New Zealand Journal of Crop and Horticultural Science*.

[13] Hammer K., Gebauer J., Al Khanjari S., Buerkert A. (2009). Oman at the cross-roads of inter-regional exchange of cultivated plants. *Genetic Resources and Crop Evolution*.

[14] Nguz K., Shindano J., Samapundo S., Huyghebaert A. (2005). Microbiological evaluation of fresh-cut organic vegetables produced in Zambia. *Food Control*.

[15] Abriouel H., Omar N. B., Molinos A. C. (2008). Comparative analysis of genetic diversity and incidence of virulence factors and antibiotic resistance among enterococcal populations from raw fruit and vegetable foods, water and soil, and clinical samples. *International Journal of Food Microbiology*.

[16] Al-Bahry S. N., Al-Zadjali M. A., Mahmoud I. Y., Elshafie A. E. (2012). Biomonitoring marine habitats in reference to antibiotic resistant bacteria and ampicillin resistance determinants from oviductal fluid of the nesting green sea turtle, *Chelonia mydas*. *Chemosphere*.

[17] Tamura K., Peterson D., Peterson N., Stecher G., Nei M., Kumar S. (2011). MEGA5: molecular evolutionary genetics analysis using maximum likelihood, evolutionary distance, and maximum parsimony methods. *Molecular Biology and Evolution*.

[18] Reasoner D. J., Geldreich E. E. (1985). A new medium for the enumeration and subculture of bacteria from potable water. *Applied and Environmental Microbiology*.

[19] Allen M. J., Edberg S. C., Reasoner D. J. (2004). Heterotrophic plate count bacteria—what is their significance in drinking water?. *International Journal of Food Microbiology*.

[20] Siragusa G. R., Dorsa W. J., Cutter C. N., Bennett G. L., Keen J. E., Koohmaraie M. (1998). The incidence of Escherichia coli on beef carcasses and its association with aerobic mesophilic plate count categories during the slaughter process. *Journal of Food Protection*.

[21] Ruimy R., Brisabois A., Bernede C. (2010). Organic and conventional fruits and vegetables contain equivalent counts of Gram-negative bacteria expressing resistance to antibacterial agents. *Environmental Microbiology*.

[22] Al-Zoreky N. S. (2009). Antimicrobial activity of pomegranate (*Punica granatum* L.) fruit peels. *International Journal of Food Microbiology*.

[23] Menezes S. M. S., Cordeiro L. N., Viana G. S. B. (2006). *Punica granatum* (pomegranate) extract is active against dental plaque. *Journal of Herbal Pharmacotherapy*.

[24] Al-Maiman S. A., Ahmad D. (2002). Changes in physical and chemical properties during pomegranate (*Punica granatum* L.) fruit maturation. *Food Chemistry*.

[25] Nhung P. H., Ohkusu K., Mishima N. (2007). Phylogeny and species identification of the family *Enterobacteriaceae* based on *dnaJ* sequences. *Diagnostic Microbiology & Infectious Disease*.

[26] Mathur S., Singh R. (2005). Antibiotic resistance in food lactic acid bacteria—a review. *International Journal of Food Microbiology*.

[27] Forslund K., Sunagawa S., Kultima J. R. (2013). Country-specific antibiotic use practices impact the human gut resistome. *Genome Research*.

[28] Abadias M., Usall J., Anguera M., Solsona C., Viñas I. (2008). Microbiological quality of fresh, minimally-processed fruit and vegetables, and sprouts from retail establishments. *International Journal of Food Microbiology*.

[29] Jonas D., Spitzmüller B., Daschner F. D., Verhoef J., Brisse S. (2004). Discrimination of *Klebsiella pneumoniae* and *Klebsiella oxytoca* phylogenetic groups and other *Klebsiella* species by use of amplified fragment length polymorphism. *Research in Microbiology*.

[30] Hamilton-Miller J. M. T., Shah S. (2001). Identity and antibiotic susceptibility of enterobacterial flora of salad vegetables. *International Journal of Antimicrobial Agents*.

[31] Brady C., Cleenwerck I., Venter S., Coutinho T., De Vos P. (2013). Taxonomic evaluation of the genus *Enterobacter* based on multilocus sequence analysis (MLSA): Proposal to reclassify *E. nimipressuralis* and *E. amnigenus* into *Lelliottia* gen. nov. as *Lelliottia nimipressuralis* comb. nov. and *Lelliottia amnigena* comb. nov., respectively, *E. gergoviae* and *E. pyrinus* into *Pluralibacter* gen. nov. as *Pluralibacter gergoviae* comb. nov. and *Pluralibacter pyrinus* comb. nov., respectively, *E. cowanii*, *E. radicincitans*, *E. oryzae* and *E. arachidis* into *Kosakonia* gen. nov. as *Kosakonia cowanii* comb. nov., *Kosakonia radicincitans* comb. nov., *Kosakonia oryzae* comb. nov. and *Kosakonia arachidis* comb. nov., respectively, and *E. turicensis*, *E. helveticus* and *E. pulveris* into *Cronobacter* as *Cronobacter zurichensis* nom. nov., *Cronobacter helveticus* comb. nov. and *Cronobacter pulveris* comb. nov., respectively, and emended description of the genera *Enterobacter* and *Cronobacter*. *Systematic and Applied Microbiology*.

[32] Kämpfer P., Glaeser S. P., Raza M. W., Abbasi S. A., Perry J. D. (2014). *Pseudocitrobacter* gen. nov., a novel genus of the *Enterobacteriaceae* with two new species *Pseudocitrobacter faecalis* sp. nov., and *Pseudocitrobacter anthropi* sp. nov, isolated from fecal samples from hospitalized patients in Pakistan. *Systematic and Applied Microbiology*.

[33] Harada H., Oyaizu H., Kosako Y., Ishikawa H. (1997). *Erwinia aphidicola*, a new species isolated from pea aphid, *Acyrthosiphon pisum*. *Journal of General and Applied Microbiology*.

[34] Marín F., Santos M., Carretero F., Yau J. A., Diánez F. (2011). *Erwinia aphidicola* isolated from commercial bean seeds (*Phaseolus vulgaris*). *Phytoparasitica*.

[35] Tash K. (2005). Rahnella aquatilis bacteremia from a suspected urinary source. *Journal of Clinical Microbiology*.

[36] Hejazi A., Falkiner F. R. (1997). *Serratia marcescens*. *Journal of Medical Microbiology*.

[37] Grohskopf L. A., Roth V. R., Feikin D. R. (2001). *Serratia liquefaciens* bloodstream infections from contamination of epoetin alfa at a hemodialysis center. *The New England Journal of Medicine*.

[38] Drelichman V., Band J. D. (1985). Bacteremias due to *Citrobacter diversus* and *Citrobacter freundii*: incidence, risk factors, and clinical outcome. *Archives of Internal Medicine*.

[39] Nada B., Areej M. (2014). *Raoultella planticola*, a central venous line exit site infection. *Journal of Taibah University Medical Sciences*.

[40] Österblad M., Pensala O., Peterzéns M., Heleniusc H., Huovinen P. (1999). Antimicrobial susceptibility of *Enterobacteriaceae* isolated from vegetables. *Journal of Antimicrobial Chemotherapy*.

[41] Gilbert R. J., de Louvois J., Donovan T. (2000). Guidelines for the microbiological quality of some ready-to-eat foods sampled at the point of sale. PHLS Advisory Committee for Food and Dairy Products. *Communicable Disease and Public Health*.

[42] Kuramoto S., Kodama H., Yamada K. (2006). Food poisoning attributable to *Staphylococcus aureus* deficient in all of the staphylococcal enterotxoin gene so far reported. *Japanese Journal of Infectious Diseases*.

[43] McGowan L. L., Jackson C. R., Barrett J. B., Hiott L. M., Fedorkacray P. J. (2006). Prevalence and antimicrobial resistance of enterococci isolated from retail fruits, vegetables, and meats. *Journal of Food Protection*.

[44] Boehm A. B., Sassoubre L. M., Gilmore M. S., Clewell D. B., Ike Y. (2014). Enterococci as indicators of environmental fecal contamination. *Enterococci: From Commensals to Leading Causes of Drug Resistant Infections*.

[45] Eaton T. J., Gasson M. J. (2001). Molecular screening of *Enterococcus virulence* determinants and potential for genetic exchange between food and medical isolates. *Applied and Environmental Microbiology*.

[46] Emori T. G., Gaynes R. P. (1993). An overview of nosocomial infections, including the role of the microbiology laboratory. *Clinical Microbiology Reviews*.

[47] Gqunta K., van Wyk J., Ekermans P., Bamford C., Moodley C., Govender S. (2015). First report of an IMI-2 carbapenemase-producing *Enterobacter asburiae* clinical isolate in South Africa. *Southern African Journal of Infectious Diseases*.

[48] Westerfeld C., Papaliodis G. N., Behlau I., Durand M. L., Sobrin L. (2009). *Enterobacter amnigenus* endophthalmitis. *Retinal Cases & Brief Reports*.

[49] Paton A. W., Paton J. C. (1996). *Enterobacter cloacae* producing a Shiga-like toxin II-related cytotoxin associated with a case of hemolytic-uremic syndrome. *Journal of Clinical Microbiology*.

[50] Wenger P. N., Tokars J. I., Brennan P. (1997). An outbreak of *Enterobacter hormaechei* infection and colonization in an intensive care nursery. *Clinical Infectious Diseases*.

[51] Khajuria A., Praharaj A. K., Grover N., Kumar M. (2013). First report of an *Enterobacter ludwigii* isolate coharboring NDM-1 and OXA-48 carbapenemases. *Antimicrobial Agents and Chemotherapy*.

[52] Iaria C., Stassi G., Costa G. B., di Leo R., Toscano A., Cascio A. (2005). Enterococcal meningitis caused by *Enterococcus casseliflavus*. First case report. *BMC Infectious Diseases*.

[53] Zeana C., Kubin C. J., Della-Latta P., Hammer S. M. (2001). Vancomycin-resistant *Enterococcus faecium* meningitis successfully managed with linezolid: Case report and review of the literature. *Clinical Infectious Diseases*.

[54] Poyart C., Lambert T., Morand P. (2002). Native valve endocarditis due to *Enterococcus hirae*. *Journal of Clinical Microbiology*.

[55] Freyaldenhoven B. S., Schlieper G., Lütticken R., Reinert R. R. (2005). *Enterococcus raffinosus* infection in an immunosuppressed patient: case report and review of the literature. *Journal of Infection*.

[56] Högenauer C., Langner C., Beubler E. (2006). *Klebsiella oxytoca* as a causative organism of antibiotic-associated hemorrhagic colitis. *The New England Journal of Medicine*.

[57] Elemam A., Rahimian J., Mandell W. (2009). Infection with panresistant *Klebsiella pneumoniae*: a report of 2 cases and a brief review of the literature. *Clinical Infectious Diseases*.

[58] Liberto M. C., Matera G., Puccio R., Lo Russo T., Colosimo E., Focà E. (2009). Six cases of sepsis caused by *Pantoea agglomerans* in a teaching hospital. *New Microbiologica*.

[59] Alves M. S., Riley L. W., Moreira B. M. (2007). A case of severe pancreatitis complicated by *Raoultella planticola* infection. *Journal of Medical Microbiology*.

[60] O' Connell K., Kelly J., NiRiain U. (2010). A rare case of soft-tissue infection caused by *Raoultella planticola*. *Case Reports in Medicine*.

[61] Equi R. A., Green W. R. (2001). Endogenous *Serratia marcescens* endophthalmitis with dark hypopyon: a case report and review. *Survey of Ophthalmology*.

[62] Tascini C., Di Paolo A., Polillo M. (2011). Case report of a successful treatment of methicillin-resistant *Staphylococcus aureus* (MRSA) bacteremia and MRSA/vancomycin-resistant *Enterococcus faecium* cholecystitis by daptomycin. *Antimicrobial Agents and Chemotherapy*.

[63] Nhung P. H., Ohkusu K., Mishima N. (2007). Phylogeny and species identification of the family *Enterobacteriaceae* based on *dnaJ* sequences. *Diagnostic Microbiology and Infectious Disease*.

[64] Rezzonico F., Smits T. H., Montesinos E., Frey J. E., Duffy B. (2009). Genotypic comparison of *Pantoea agglomerans* plant and clinical strains. *BMC Microbiology*.

[65] Spröer C., Mendrock U., Swiderski J., Lang E., Stackebrandt E. (1999). The phylogenetic position of *Serratia, Buttiauxella* and some other genera of the family *Enterobacteriaceae*. *International Journal of Systematic Bacteriology*.

[66] Takahashi T., Satoh I., Kikuchi N. (1999). Phylogenetic relationships of 38 taxa of the genus *Staphylococcus* based on 16S rRNA gene sequence analysis. *International Journal of Systematic Bacteriology*.

[67] Naser S. M., Thompson F. L., Hoste B. (2005). Application of multilocus sequence analysis (MLSA) for rapid identification of *Enterococcus* species based on *rpoA* and *pheS* genes. *Microbiology*.

